# Design and Development of a Big Data Platform for Disease Burden Based on the Spark Engine

**DOI:** 10.1155/2023/8963053

**Published:** 2023-02-06

**Authors:** Chengcheng Li, Jing Gao, Qingwei Pan, Zhihua Zhou, Yue Yang, Shangcheng Zhou

**Affiliations:** ^1^School of Public Health and Management, Guangzhou University of Chinese Medicine, Guangzhou 510006, China; ^2^College of Physical Education and Health, Guangxi Medical University, Nanning 530021, China; ^3^School of Life Science and Technology, Inner Mongolia University of Science and Technology, Baotou 014010, China

## Abstract

**Objective:**

This study attempts to build a big data platform for disease burden that can realize the deep coupling of artificial intelligence and public health. This is a highly open and shared intelligent platform, including big data collection, analysis, and result visualization.

**Methods:**

Based on data mining theory and technology, the current situation of multisource data on disease burden was analyzed. Putting forward the disease burden big data management model, functional modules, and technical framework, Kafka technology is used to optimize the transmission efficiency of the underlying data. This will be an efficient and highly scalable data analysis platform through embedding embedded Sparkmlib in the Hadoop ecosystem.

**Results:**

With the concept of “Internet + medical integration,” the overall architecture design of the big data platform for disease burden management was proposed based on the Spark engine and Python language. The main system composition and application scenarios are given at four levels: multisource data collection, data processing, data analysis, and the application layer, according to application scenarios and use requirements.

**Conclusion:**

The big data platform of disease burden management helps to promote the multisource convergence of disease burden data and provides a new path for the standardized paradigm of disease burden measurement. Provide methods and ideas for the deep integration of medical big data and the formation of a broader standard paradigm.

## 1. Background

The explosive prosperity of Internet of things technology and artificial intelligence has promoted profound changes in the industry worldwide [1]. Information resources are gradually becoming one of the most essential strategic resources. This large-scale, fast-growing, diverse data structure and multidimensional value density data set is often called big data [2]. How to identify and extract the key features of information in a timely, accurate, and efficient manner from the data ocean and conduct rapid analysis has become one of the hot issues [3, 4].

There are many positive explorations in the construction of big data platforms in many fields around the world. A study from Spain explored the construction of a social media big data platform, which can monitor early signs of depression in 2020 [5]. In the field of transportation, some scholars have realized the dynamic benefits of monitoring of traffic violations through a big data platform [6]. Moreover, the education big data platform achieved the informational management of student courses and effectively improved the teaching quality in the field of education [7]. Some scholars have built a big data platform based on the lambda architecture and applied it in the energy field to realize the visual monitoring of residential power consumption and effectively improve energy utilization efficiency [8]. Not only that, but the big data platform based on the lambda architecture can also dynamically monitor and analyze marine activities and integrate various parameters [9]. In addition to it, cloud computing technology provides new solutions and computing potential for the storage and management of large-scale data, relying on the scientific Earth big data platform [10].

Big data has wider application scenarios and development potential in the field of medical research. The United States promotes the opening and sharing of big data to the medical industry to realize technological innovation. This is a strategic plan to encourage multinational pharmaceutical enterprises to deeply explore the hidden application value in the massive medical information by implementing the “big data research and development plan” [11]. On this basis, multinational pharmaceutical enterprises try to achieve accurate drug development by deeply integrating big data technology with precision medicine [12]. These highly personalized precision treatment schemes have provided great help to further complete the “human genome project” [13]. In 2015, the United States proposed to establish a global open cancer genomics database and named it the “moon landing project” for cancer [14]. The United States has realized the construction of local full-coverage medical big data through 12 electronic case data centers, 9 medical data centers, and 8 biological information databases in 2018 [15]. Meanwhile, Korean doctors have obtained a large amount of data to implement precision medicine by creating a big data platform for cancer [16]. The application of big data medical platforms can reduce neonatal mortality and disability rates effectively [17]. This is a rapidly developing trend in medical big data research around the world.

China has introduced a series of favorable policies to promote technological development and the industrial landing of big data. In 2016, China issued guiding opinions on promoting and standardizing the application and development of big data in health care, aiming to promote the “healthy China 2030” plan to realize the medical mode of “Internet +” for the whole industry chain [18]. Meanwhile, policymakers respond to national strategic needs by innovating the talent training mode and strengthening the talent training of cross-disciplinary fields such as artificial intelligence and big data. In the “13th five-year plan” for the development of national population health informatization in 2017, it is clearly pointed out that the health care big data service system in 2021, which is composed of regional medical big data centers and supporting clinical data analysis centers, will be initially established and issued by the China Health Commission [19]. The “14th five-year plan” for national informatization in 2021 further emphasizes the exploration of the application effect of artificial intelligence in intelligent clinical assistant diagnosis and treatment, intelligent public health service, and artificial intelligence-assisted drug research and development, and emphasizes the key role of the construction of a scientific research big data platform in the development of big data [20]. Codelot, the national life big data platform, was established in Shenzhen with strong policy support and can provide various functions, including gene detection, biological information analysis, and data mining. Chinese scholars have developed a medical imaging platform that relies on cloud computing technology to enhance the diagnostic efficiency of obstetric imaging [21]. The medical big data platform has also played an active role in the rapid gene diagnosis of the digestive department in clinical practice [22].

Disease burden data has the characteristics of high capacity, rapid growth, diverse types, and low value density [23]. Some scholars have examined a lot of high-quality research in the field of disease burden [24–26]. Therefore, the technological breakthrough of big data in disease burden research is very important. At present, the research on disease burden focuses more on manual analysis using machine learning algorithms. Of course, there is a lack of research on the intelligent extraction of massive medical data [27]. At present, there are few unified big data platforms for disease burden research. Hadoop architecture provides us with inspiration and ideas for our research based on Java and Python languages [28].

This study attempts to solve the following problems from the perspective of artificial intelligence: (1) the big data platform for disease burden is designed and created based on the Hadoop distributed computing framework. (2) Optimize the calculation efficiency and accuracy based on the Spark engine in the traditional distributed computing framework. (3) We try to realize the visual design of the analysis results in order to provide an analysis paradigm for the related research on disease burden in the future.

## 2. Methods

This study attempts to build a big data platform for disease burden analysis based on the Spark engine, which can achieve the whole process of data dynamic capture, storage, analysis, detection, and visual output in disease burden analysis. It can further optimize the efficiency of machine learning algorithms by embedding Spark into Hadoop [29, 30]. There is a wide gap between Hadoop and Spark in the actual construction process, as shown in Table 1.

There are many similarities between Spark and Hadoop, both of which are open-source cluster computing environments. We call it Sparkmlib to realize real-time calculation, access tracking, and anomaly detection of disease burden data.

### 2.1. Workflow of Spark Streaming

**Figure 1:**
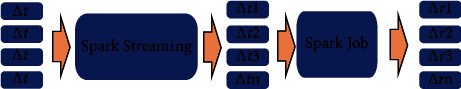
Workflow of Spark streaming.

A discretized stream is the data over time, and it is also a sequence composed of the RDD of each time interval [31]. DStreams will be formed from multiple input sources such as Flume, Kafka, or HDFS, as shown in Figure 1.

Specifically, Spark streaming treats streaming computing as a series of continuous small-scale batch processing. Data will be divided into small batches by reading data from different input sources, with the creation of new batches occurring at uniform intervals. A new batch will be formed at the beginning of each time interval. The data received during the interval will be added to the batch. The batch will stop growing at the end of the time interval.

### 2.2. Big Data Measurement Paradigm of Disease Burden

**Figure 2:**
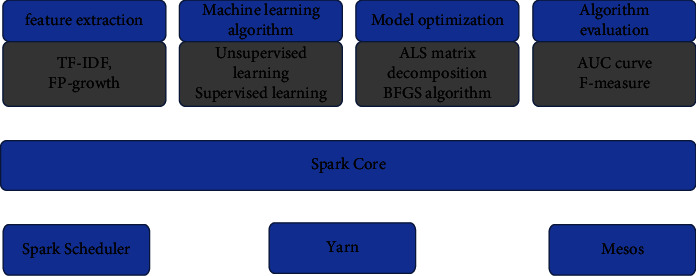
Big data measurement paradigm based on Spark engines.

The design of the mlib algorithm package based on Spark is simple [32]. First, the data is expressed in the form of an RDD, and then various algorithms are called on the distributed dataset. We try to embed JOINTPOINT software and DISMOD software into our disease burden big data platform. We have built a whole process big data analysis platform based on Spark engine in this study. The algorithm is shown in Figure 2.

Apache Hadoop Yarn is an ideal Hadoop resource manager [33]. As a general resource management system, it can uniformly schedule applications on the platform. The advantages of strong compatibility can bring great benefits to the cluster. Resource management and scheduling are realized by creating application managers and global managers of MapReduce traditional applications for HDFS [34]. As a supplement to MapReduce, hive improves the ability for rapid development of big data platforms and reduces the difficulty of building big data platforms through SQL-like syntax [35].

**Figure 3:**
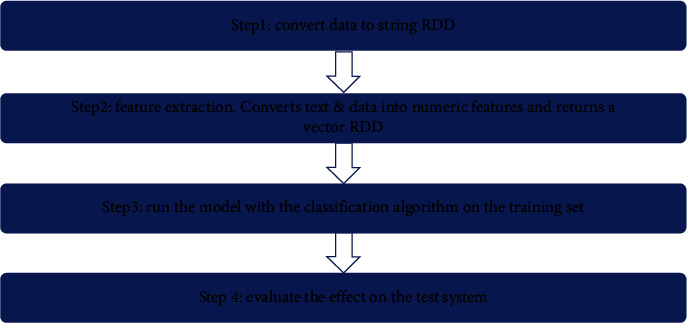
Big data analysis steps.

In general, we split the big data analysis algorithm into the following four steps, as shown in Figure 3.

### 2.3. Feature Extraction Algorithm

#### 2.3.1. TF-IDF Algorithm

TF-IDF (term frequency-inverse document frequency) is a classic weighting technique for information retrieval and text mining [36].

TF represents the probability of a keyword's occurrence in the text. Normalization can prevent deviations in text mining. The formula is as follows:

(1) $$\begin{array}{c} tf_{ij}=\frac{n_{i,j}}{\sum_{k}n_{i,j}}\text{,} \end{array}$$

where *n*_*i*,*j*_ is the number of times the word appears in the file *i*, *j*.

Furthermore, this term can have an excellent ability to distinguish categories in the case of fewer documents of *T* and large IDF. The calculation method is as follows:

(2) $$\begin{array}{c} idf_{i}=\log\frac{\left|D\right|}{\left|D\left\{j:t_{i}\in d_{j}\right\}\right|}\text{,} \end{array}$$

where |*D*| is the entire number of files in the corpus. {*j* : *t*_*i*_ ∈ *d*_*j*_} denotes the number of files containing the word *t*_*i*_ (i.e., the number of files with *n*_*i*_, *j* ≠ 0). Usually, 1+|{*j* : *t*_*i*_ ∈ *d*_*j*_}| is used to avoid the case that the word is not in the corpus and the denominator is 0. Actually, TF-IDF is better at filtering out common words while retaining important words. The calculation method is as follows:

(3) $$\begin{array}{c} TF-IDF=TF*IDF\text{.} \end{array}$$

#### 2.3.2. FP Growth Algorithm

**Figure 4:**
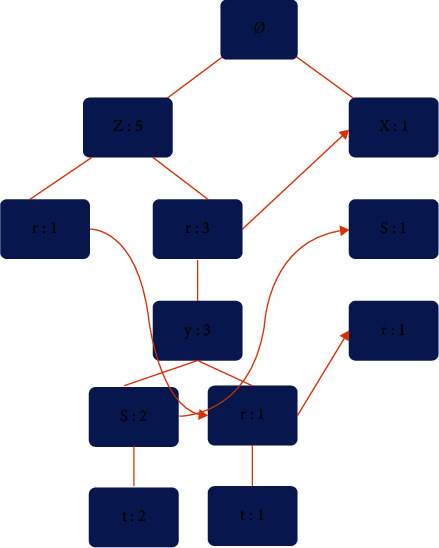
FP tree based on certain data structures.

In order to further optimize the keyword screening strategy, we tried to introduce a FP growth algorithm based on the TF-IDF algorithm. [37] This algorithm finds and recommends high-frequency word pairs by looking at the words used on the Internet. The data consistency of medical electronic cases is weak due to the wide range of sources. Therefore, the FP growth algorithm can comprehensively extract and collect data. The algorithm logic is as follows:

First, building an FP Tree based on a certain data structure, as shown in Figure 4.

FP Tree is not used for simple decision trees, so a class should be created to save each node of the tree. The FP Tree will store the occurrence frequency of item sets. Only when the sets are completely different, the tree will fork.

Second, the conditional pattern library is obtained from the FP Tree, and frequent item sets are mined so as to build a larger set on the basis of a single element itemset. This is an effective way to create a conditional FP Tree, which can repeatedly cycle other single-element items for each frequent item.

## 3. Model Optimization Algorithm

### 3.1. ALS (Alternating Least Squares) Algorithm

The Spark platform integrates the ALS algorithm. The optimization of matrix decomposition can be realized quickly by constructing different loss functions [38]. The final task of matrix decomposition is to find two matrices, *P* and *Q*, and make them approximately equal to the original matrix *R* after multiplication. The specific algorithm is as follows:

(4) $$\begin{array}{c} R_{m*n}=P_{m*k}\times Q_{n*k}^{T}\text{,} \end{array}$$

where *P* and *Q* are unknown. We assume that *Q* is known. Therefore,

(5) $$\begin{array}{c} P_{m*k}=R_{m*n}\times Q_{n*k}^{-1}\text{.} \end{array}$$

This means that the *R* matrix is multiplied by the inverse matrix of the *Q* matrix, and the result is obtained through iteration. It is assumed that the solving process is carried out alternately until the error is acceptable.

### 3.2. L-BFGS Optimization Algorithm

This optimization algorithm evolved from the Newton method and the quasi-Newton method and has been widely used commercially [39]. The specific algorithm is as follows:

Let *f*(*x*) be a quadratic differentiable real function, set up again *χ*^(*k*)^ is an estimate of a minimal point of *f*(*x*). We expand *f*(*x*) into Taylor series at *χ*^(*k*)^ and take the second-order approximation.

(6) $$\begin{array}{c} f\left(x\right)\approx \emptyset \left(x\right)=f\left(x^{\left(k\right)}\right)+{\nabla f\left(x^{\left(k\right)}\right)}^{T}\left(x-x^{k}\right)+\frac{1}{2}{\left(x-x^{\left(k\right)}\right)}^{T}\nabla^{2}f\left(x^{\left(k\right)}\right)\left(x-x^{\left(k\right)}\right)\text{,} \end{array}$$

where the middle part of the last item present Hesse matrix of *f*(*x*) at *x*^(*k*)^. The following formula can be obtained by deriving the abovementioned formula and making it equal to 0:

(7) $$\begin{array}{c} \nabla f\left(x^{\left(k\right)}\right)+\nabla^{2}f\left(x^{\left(k\right)}\right)\left(x-x^{k}\right)=0\text{.} \end{array}$$

Assuming that the Hesse matrix is reversible, the iterative formula of the Newton method can be obtained from the abovementioned formula as follows:

(8) $$\begin{array}{c} x^{\left(k+1\right)}=x^{\left(k\right)}-\nabla^{2}{f\left(x^{\left(k\right)}\right)}^{-1}\nabla f\left(x^{\left(k\right)}\right)\text{,} \end{array}$$

(9) $$\begin{array}{c} x^{\left(k+1\right)}=x^{\left(k\right)}+\lambda_{k}d^{\left(k\right)}\text{,} \end{array}$$

(10) $$\begin{array}{c} d^{\left(k\right)}=-\nabla^{2}{f\left(x^{\left(k\right)}\right)}^{-1}\nabla f\left(x^{\left(k\right)}\right)\text{,} \end{array}$$

where *λ* is the compensation obtained by one-dimensional search, which means

(11) $$\begin{array}{c} f\left(x^{\left(k\right)}+\lambda_{k}d^{\left(k\right)}\right)=minfx^{\left(k\right)}+\lambda_{k}d^{\left(k\right)}\text{.} \end{array}$$

We try to construct the approximate matrix of the inverse matrix of the Hesse matrix by analyzing the association between the inverse matrix and the first derivative. Assume that *χ*^(*k*+1)^ is obtained after the *k*-th iteration. We expand the objective function *f* (*x*) into the Taylor series at point *χ*^(*k*+1)^ and take the second-order approximation to obtain

(12) $$\begin{array}{c} f\left(x\right)\approx \left.{f\left(x\right.}^{\left(k+1\right)}\right)+\nabla f{\left(x^{\left(k+1\right)}\right)}^{T}\left(x\right.-\left.x^{k}\right)+\frac{1}{2}{\left(x-x^{\left(k+1\right)}\right)}^{T}\nabla^{2}f\left(x^{\left(k+1\right)}\right)\left(x-x^{\left(k+1\right)}\right)\text{.} \end{array}$$

It can be seen that in the vicinity of *χ*^(*k*+1)^,

(13) $$\begin{array}{c} \nabla f\left(x\right)\approx \nabla f\left(x^{k+1}\right)+\nabla^{2}f\left(x^{k+1}\right)\left(x-x^{\left(k+1\right)}\right)\text{,} \end{array}$$

(14) $$\begin{array}{c} \nabla f\left(x^{\left(k\right)}\right)\approx \nabla f\left(x^{k+1}\right)+\nabla^{2}f\left(x^{k+1}\right)\left(x^{\left(k\right)}-x^{\left(k+1\right)}\right)\text{,} \end{array}$$

(15) $$\begin{array}{c} p^{\left(k\right)}=x^{\left(k+1\right)}-x^{\left(k\right)}\text{,} \end{array}$$

(16) $$\begin{array}{c} q^{\left(k\right)}=\nabla f\left(x^{\left(k+1\right)}\right)-\nabla f\left(x^{\left(k\right)}\right)\text{,} \end{array}$$

(17) $$\begin{array}{c} q^{\left(k\right)}\approx \nabla^{2}f\left(x^{\left(k+1\right)}\right)p^{\left(k\right)}\text{,} \end{array}$$

(18) $$\begin{array}{c} p^{\left(k\right)}\approx \nabla^{2}f\left(x^{\left(k+1\right)}\right)q^{\left(k\right)}\text{,} \end{array}$$

(19) $$\begin{array}{c} p^{\left(k\right)}=H_{k}q^{\left(k\right)}\text{.} \end{array}$$

Therefore, formula (19) is called the quasi-Newton condition.

When the inverse matrix of the Hesse matrix is a symmetric positive definite matrix, the matrix *H*_(*K*)_ satisfying the quasi-Newton condition should also be a symmetric positive definite matrix. We assume that

(20) $$\begin{array}{c} H_{k+1}=H_{k}+∆H_{k}\text{,} \end{array}$$

(21) $$\begin{array}{c} H_{k+1}=H_{k}+\frac{\left(p^{\left(k\right)}-H_{k}q^{\left(k\right)}\right){\left.{\left(p\right.}^{\left(k\right)}-H_{k}q^{\left(k\right)}\right)}^{T}}{q^{\left(k\right)T}\left(p^{\left(k\right)}-H_{k}q^{\left(k\right)}\right)}\text{.} \end{array}$$

Then, we define that

(22) $$\begin{array}{c} ∆H_{k}=\frac{p^{\left(k\right)}p^{\left(k\right)T}}{p^{\left(k\right)T}q^{\left(k\right)}}-\frac{H_{k}q^{\left(k\right)}q^{\left(k\right)T}H_{k}}{q^{\left(k\right)T}H_{k}q^{\left(k\right)}}\text{,} \end{array}$$

(23) $$\begin{array}{c} H_{k+1}=H_{k}+\frac{p^{\left(k\right)}p^{\left(k\right)T}}{p^{\left(k\right)T}q^{\left(k\right)}}-\frac{H_{k}q^{\left(k\right)}q^{\left(k\right)T}H_{k}}{q^{\left(k\right)T}H_{k}q^{\left(k\right)}}\text{.} \end{array}$$

We swap *H* equals *B*, *p* and *q*; therefore,

(24) $$\begin{array}{c} q^{\left(k\right)}=B_{k+1}p^{\left(k\right)}\text{,} \end{array}$$

(25) $$\begin{array}{c} B_{k+1}=B_{k}+\frac{q^{\left(k\right)}q^{\left(k\right)T}}{q^{\left(k\right)T}p^{\left(k\right)}}-\frac{B_{k}p^{\left(k\right)}p^{\left(k\right)T}B_{k}}{P^{\left(k\right)T}B_{k}p^{\left(k\right)}}\text{.} \end{array}$$

We assume that *B*_(*K*+1)_ is reversible, then,

(26) $$\begin{array}{c} H_{k+1}=B_{k+1}^{-1}\text{.} \end{array}$$

Finally, the BFGS formula for *H* was obtained as

(27) $$\begin{array}{c} H_{k+1}^{BFGS}=H_{k}+\left(1+\frac{q^{\left(k\right)T}{H_{k}q}^{\left(k\right)}}{p^{\left(k\right)T}q^{\left(k\right)}}\right)\frac{p^{\left(k\right)}p^{\left(k\right)T}}{p^{\left(k\right)T}q^{\left(k\right)}}-\frac{p^{\left(k\right)}q^{\left(k\right)T}H_{k}+H_{k}q^{\left(k\right)}p^{\left(k\right)T}}{p^{\left(k\right)T}q^{\left(k\right)}}\text{.} \end{array}$$

The iteration of the *D*-matrix can be realized through iterative calculation. Furthermore, storage space can be effectively saved by transforming the matrix into a vector. Therefore, the algorithm convergence process for big data can be realized by another approximation of the BFGS algorithm, which is also called the L-BFGS algorithm.

### 3.3. Validation of the Model

The accuracy of the model can be effectively verified through the accuracy test. Accuracy, classification error rate, precision, recall, and *F*1_score were the five indicators of score used to evaluate the effectiveness of amchine learning algorithms. The specific formulas are as follows:

(28) $$\begin{array}{c} Accuracy=\frac{TP+TN}{TP+TN+FP+FN}\text{,} \end{array}$$

(29) $$\begin{array}{c} Classification\;error\;rate=1-accuracy\text{,} \end{array}$$

(30) $$\begin{array}{c} Preciion\left(p\right)=\frac{TP}{\left(TP+FP\right)}\text{,} \end{array}$$

(31) $$\begin{array}{c} Recall\left(R\right)=\frac{TP}{\left(TP+FN\right)}\text{,} \end{array}$$

(32) $$\begin{array}{c} F1score=\frac{2*\left(P*R\right)}{\left(P+R\right)}\text{,} \end{array}$$

where TP means true positive; TN means true negative; FP means false positive; FN means false negative. In the *F*1_ score calculation formula, precision is abbreviated as *P*, and recall is abbreviated as *R*. The *F*1_score value ranges from 0 to 1, with 1 indicating the best and 0 the worst.

## 4. Results

### 4.1. Big Data Platform Construction Framework

**Figure 5:**
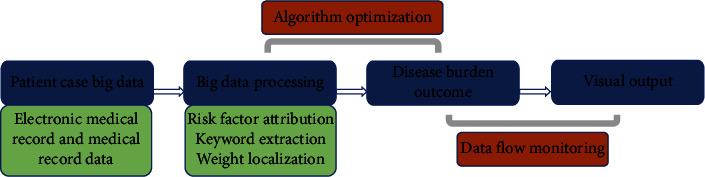
Disease burden big data cloud platform architecture.

The big data infrastructure is a stack-type technology architecture [40], Mainly including the following: (1) the foundation layer; (2) the management level: not only the storage and management of existing data, but also the calculation of some data; (3) the analysis layer: embed the corresponding statistical model and machine learning algorithm to analyze the data according to the research objectives; (4) application layer: mainly for the user's front-end development and visual output, as shown in Figure 5.

### 4.2. Storage Engine

**Figure 6:**
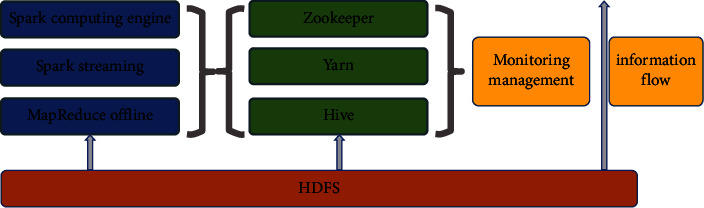
Organizational framework and construction idea of the foundation layer.

As the bottom layer of the big data architecture of disease burden, the analysis layer is included in the platform building framework of the basic layer in this study. The big data platform building ideas of Internet enterprises are used for reference and optimized to form a highly automated computing platform that can be expanded horizontally. Specifically, the Kudu storage engine is used for large-scale data storage to balance the performance of HDFS and HBase random reading and writing and batch analysis [41]. The access to a remote MySQL database is implemented by Faderated, and on this basis, the basic layer of the big data platform for estimating disease burden is built, as shown in Figure 6.

### 4.3. Big Data Operation

**Figure 7:**
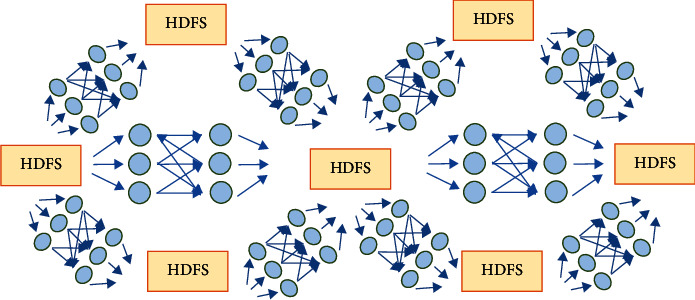
MapReduce data processing process.

After the storage engine is selected, since the traditional Hadoop ecosystem cannot complete the ETL and data cleaning work in one MapReduce, the data calculation and processing are realized through the hybrid construction of Spark and MapReduce, as shown in Figure 7.

**Figure 8:**
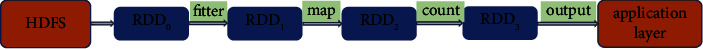
Elastic distributed data set framework.

In order to improve the calculation efficiency and reduce the fault tolerance of the distributed system, the Spark process builds an elastic distributed data set by referring to the idea of functional programming. As a read-only and partitioned data set, RDD forms a directed acyclic graph through operator connection, which significantly improves the computational efficiency. The transformation between various operators is realized through stream, as shown in Figure 8.

Therefore, Spark, MapReduce, and Sparkstreaming jointly complete the cloud computing process of the disease burden big data platform. The sorting and coordination of data resources are jointly completed by Zookeeper and Apache Hadoop Yarn. The zookeeper server processes the client's request through FIFO, allowing the client to connect to any subserver and providing higher performance.

### 4.4. Multisource Data Acquisition, Cleaning, and Integration

For the construction of the big data platform for disease burden, the management level should unify the management and identification of structured data and unstructured data. Meanwhile, the real-time data should have the ability for rapid query and error identification, and the system response time should be shortened as much as possible. At the same time, sufficient operable space should be provided for the future upgrading of the system.

Therefore, the data types are mainly divided into three categories, mainly including the following:

1. Front-end logs: big data from the Internet, medical and health institutions, and mobile phones;
2. Back-end log: summarize and transmit data from subservers around the world;
3. data from MySQL database of public security, civil affairs and other institutions.

**Figure 9:**
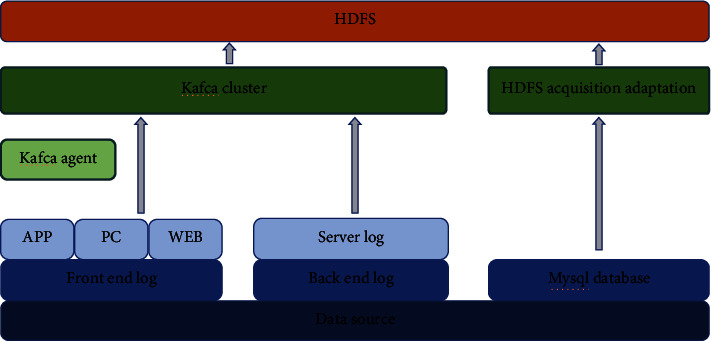
Data acquisition framework.

For different types of data flows, the Kafka producer protocol is implemented based on Lua to achieve efficient data collection. The specific framework is shown in Figure 9.

### 4.5. Call of Log Data

**Figure 10:**
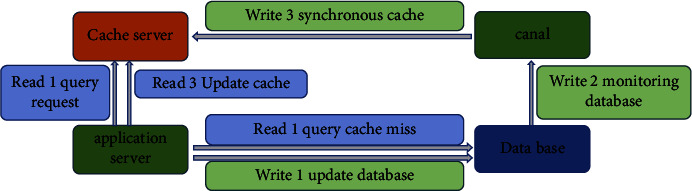
MySQL database data collection optimization strategy.

For the front-end log collection and access, it is required to have high reliability and availability while responding in real time [42]. By referring to the construction ideas and failure cases of Internet enterprises, a large number of tests have compared Flume, Scribe and Chukwa's various construction ideas and frameworks, which cannot meet the collection and storage of super-large amounts of disease burden data. Therefore, a set of data acquisition gateway can be developed based on Kafka to complete data acquisition and realize through nginx Lua. The back-end log collection and access can use Go language to realize file collection because the server logs are relatively stable. At the same time, multiple reconfigurations and optimizations can be carried out according to the needs of future research. The traditional database collection and access method uses canal to update the cache, which leads to slow MySQL query speed and no QPS. It is easy to cause paralysis if a large number of requests are sent to MySQL. Therefore, we proposed the solution of adding a cache in front of MySQL during the construction, as shown in Figure 10.

Specifically, when the cache is exhausted, MySQL will write another copy to the cache. When the data is inconsistent (MySQL database changes), modify it asynchronously, and then start a canal service to monitor MySQL to make the synchronous cache consistent.

### 4.6. Application Layer Construction

In the construction of the application layer, we mainly highlight two functions. The first is the real-time monitoring of various types of data streams on the Internet and the stability monitoring of front-end log data streams. The second is the visual presentation of disease burden measurement results. By using AI to design the UI interface for the visual output of disease burden results, we hope to improve the big data analysis ability of disease burden in China. The elk monitoring system has the following advantages:

- Business data analysis: collect key steps from the front-end information to the back-end for business process analysis.
- Error log analysis: similar to bugly, after the error log is reported, errors can be summarized, displayed by category, and analyzed in the back end.
- Data early warning: with elk, it is easy to establish an early warning mechanism for monitoring fields and conduct early warning before large-scale errors occur.

### 4.7. Visual Output of Disease Burden Results

**Figure 11:**
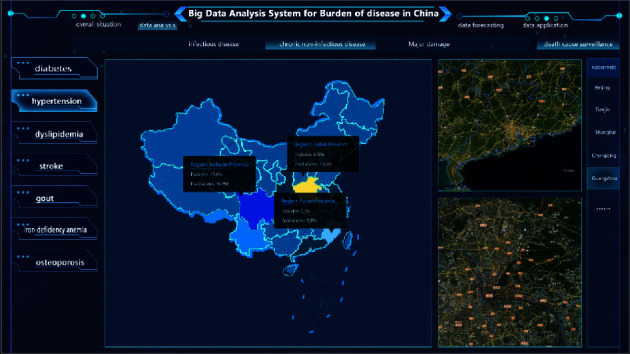
Overall overview of China's disease burden big data analysis system.

**Figure 12:**
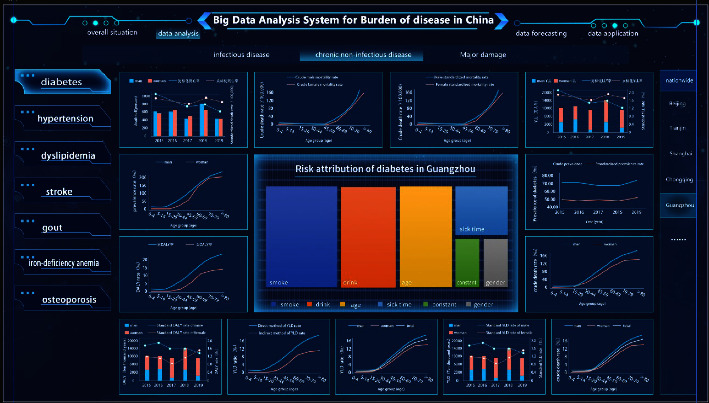
Data analysis module of China disease burden big data analysis system.

The results of the analysis of disease burden are visually output through the UI interface. Specifically, it includes four modules: an overall overview, data analysis, data prediction, and data application (Figures 11 and 12). This study designed a visual display of a big data platform based on the calculation results of the burden of diabetes in Guangzhou, China. In the overall overview module, Baidu Map is embedded in the whole disease analysis big data platform, which can realize real-time tracking and analysis of data and visually output the overall situation of diseases according to different disease classifications.

In the data analysis module, the disease burden is mainly measured and demonstrated by big data. Based on the research on regional disease burden, a big data analysis system for urban disease burden has been formed. We try to form an integrated solution from data collection to analysis to decision-making.

## 5. Conclusion

Medical and health data, especially the massive microdata in the measurement of disease burden, have the characteristics of complex, multisource, and diverse data. At the same time, it also has a complexity and diversity different from other data types, which often makes medical workers and health managers unable to use conventional software tools to acquire, manage, and integrate medical and health data in a short time, making it valuable information. It is particularly important to use big data technology to solve this problem. The construction of the big data platform for disease burden is different from that of enterprises and businesses. Since the service objects of the big data platform are mainly health department decision-makers and relevant researchers, what application layer can display the analysis results most intuitively and objectively? Can the disease burden analysis results on the IHME website be further improved to enhance readability? Domestic direct research on the construction of a health big data platform is relatively rare, and more research focuses on theoretical research and empirical measurement.

A large number of big data enterprise-level application practices have proved that the disease burden big data platform based on Spark engine can effectively realize the collection and intelligent management of multisource heterogeneous medical data. On the basis of the traditional distributed computing framework, it has greatly optimized and improved different links and levels, especially in the distributed computing of big data, which has high application value and practical significance in this field. In the future, we can apply big data technology to build a unified information management platform and strengthen the construction of data set standards, technical standards, and data sharing and exchange standards, so as to realize the effective application of medical and health data information and promote data integration and information sharing.

There are some shortcomings in this study. (1) Due to the updating of technology, the convergence and quasi-combination of the algorithm do not reach the optimal solution. At the same time, the degree of matching of different data types to the algorithm is not nearly the same, which needs more detailed research in the future. (2) Some prediction algorithms are still under active design and development and are not included in the design framework of the current big data platform. (3) Due to the heavy programming workload required for platform construction, the big data analysis platform built in this study is still under active construction and has not been put into use. The platform will be built and put into use in the future.

## Figures and Tables

**Table 1:** Technical differences between Spark and Hadoop.

	Hadoop	Spark
Type	Basic platform, including calculation, storage, and scheduling	Pure distributed computing tools
Scene	Mass data batch processing (disk iterative calculation)	Massive data batch processing (memory iterative calculation, interactive calculation), massive data stream calculation
Price	Low	High
Programming paradigm	MAP + REDUCE	RDD is a DAG directed acyclic graph
	API level is relatively low, and algorithm adaptability is poor	The API is top-level and easy to use

Data storage structure	The calculation result is on the HDFS disk with a large delay	RDD intermediate operation results are stored in memory with a small delay
Operation mode	Tasks are maintained in process mode, and the task starts slowly	Tasks are maintained in a threaded manner, with fast task startup, and can be created in batch to improve the parallel ability

## Data Availability

The data used to support the findings of this study are available from the corresponding author upon request.
